# A Critical Review on Human Malaria and Schistosomiasis Vaccines: Current State, Recent Advancements, and Developments

**DOI:** 10.3390/vaccines11040792

**Published:** 2023-04-04

**Authors:** Arif Jamal Siddiqui, Jyoti Bhardwaj, Juhi Saxena, Sadaf Jahan, Mejdi Snoussi, Fevzi Bardakci, Riadh Badraoui, Mohd Adnan

**Affiliations:** 1 Department of Biology, College of Science, University of Ha’il, Ha’il P.O. Box 2440, Saudi Arabia; 2 Division of Infectious Diseases, Department of Medicine, Indiana University School of Medicine, Indianapolis, IN 46202, USA; 3 Department of Biotechnology, University Institute of Biotechnology, Chandigarh University, Gharuan, NH-95, Ludhiana—Chandigarh State Hwy, Mohali 140413, India; 4 Department of Medical Laboratory Sciences, College of Applied Medical Sciences, Majmaah University, Al Majmaah 11952, Saudi Arabia; 5 Laboratory of Genetics, Biodiversity and Valorization of Bio-Resources (LR11ES41), Higher Institute of Biotechnology of Monastir, University of Monastir, Avenue TaharHaddas BP74, Monastir 5000, Tunisia; 6 Section of Histology-Cytology, Medicine Faculty of Tunis, University of Tunis El Manar, Tunis 1017, Tunisia

**Keywords:** malaria, schistosomiasis, vaccine, RTS,S/AS01, R21/Matrix-M, Sm-p80, clinical trial, antigen

## Abstract

Malaria and schistosomiasis are two major parasitic diseases that remain leading causes of morbidity and mortality worldwide. Co-infections of these two parasites are common in the tropics, where both diseases are endemic. The clinical consequences of schistosomiasis and malaria are determined by a variety of host, parasitic, and environmental variables. Chronic schistosomiasis causes malnutrition and cognitive impairments in children, while malaria can cause fatal acute infections. There are effective drugs available to treat malaria and schistosomiasis. However, the occurrence of allelic polymorphisms and the rapid selection of parasites with genetic mutations can confer reduced susceptibility and lead to the emergence of drug resistance. Moreover, the successful elimination and complete management of these parasites are difficult due to the lack of effective vaccines against *Plasmodium* and *Schistosoma* infections. Therefore, it is important to highlight all current vaccine candidates undergoing clinical trials, such as pre-erythrocytic and erythrocytic stage malaria, as well as a next-generation RTS,S-like vaccine, the R21/Matrix-M vaccine, that conferred 77% protection against clinical malaria in a Phase 2b trial. Moreover, this review also discusses the progress and development of schistosomiasis vaccines. Furthermore, significant information is provided through this review on the effectiveness and progress of schistosomiasis vaccines currently under clinical trials, such as Sh28GST, Sm-14, and Sm-p80. Overall, this review provides insights into recent progress in malarial and schistosomiasis vaccines and their developmental approaches.

## 1. Introduction

Malaria and schistosomiasis are both endemic parasitic diseases, causing significant morbidity and deleterious socioeconomic impacts. Schistosomiasis is the second most common parasitic disease after malaria, and both share overlapping geographic distribution patterns and high co-infection rates in the tropics and subtropics [1]. Children have the highest malaria prevalence, mainly caused by *Plasmodium falciparum* (*P. falciparum*) (Welch, 1897) [2]. The probability and dominance of malaria and schistosomiasis co-infection are very high, especially in fishing villages, which are a target population for schistosomiasis vaccine studies due to high malaria and schistosomiasis transmission and extensive exposure [3]. Because of the widespread breeding opportunities in the lake environment, large vector populations are maintained in these areas, such as stagnant pools along shorelines, abandoned old boats, fish bait mines, and finger pond traps [4]. In addition, fishing practices such as night fishing and temporary housing, which are amenable to the malaria vector, also encourage external transmission [5].

An obligate parasite of insects and vertebrates, *Plasmodium* is a genus of unicellular eukaryotes. Malaria is a serious public health problem affecting largely the tropical and subtropical regions of the world. It is mainly transmitted by parasites of the different *Plasmodium* species [6]. The female *Anopheles* mosquito, which transmits malaria to humans, is a serious health threat. Five *Plasmodium* species (*P. falciparum*, *P. malariae* (Grassi et Feletti, 1890), *P. vivax* (Grassi et Feletti, 1890), *P. ovale* (Stephens, 1922), and *P. knowlesi* (Franchini, 1927)) have long been known to infect humans and cause disease, although there are more than 100 *Plasmodium* species that can infect numerous animal species [7]. More than 90% of malaria deaths worldwide are caused by infection with *P. falciparum* and *P. vivax*; however, *P. falciparum* is responsible for the high disease burden in sub-Saharan Africa and Asia [8]. The effective and broad implementation of malaria control measures, including rapid diagnostic testing, highly effective artemisinin combination therapy, insecticide-treated bed nets, and indoor residual spraying, has substantially reduced the malaria burden and the associated morbidity and mortality. However, progress has stalled over the last ~8 years. The latest statistics show that there were around 241 million malaria cases worldwide in 2020, with almost 627,000 deaths [9], underscoring the need for a highly effective malaria vaccine.

The exchange between the primary host (mosquito) and the secondary host (human) is a feature of the *Plasmodium* life cycle. *Plasmodium* infection starts when an infected female *Anopheles* mosquito bites a person and injects an infectious form of the parasite, called sporozoites, into the bloodstream. The sporozoites quickly reach the human liver and initiate the pre-erythrocytic stage of malaria. In the liver, the parasite multiplies asexually and gives rise to the first generation of merozoites [10,11]. The released merozoites invade human red blood cells (RBCs), undergo erythrocytic schizogony, and progress through the stages of ring, trophozoite, and schizont, which rupture, releasing daughter merozoites that re-infect new erythrocytes. Some infected blood cells leave the cycle of asexual multiplication and differentiate into the sexual form of the parasite, called gametocytes. These circulating male and female gametocytes are ingested by a feeding mosquito, where they undergo the sporogonic cycle and generate sporozoites. Thus, the *Plasmodium* life cycle is a progression of differentiation stages, each characterized by the expression of proteins unique to that stage and some immune system targets in the host [12]. This makes the rational design of an ideal vaccine more challenging for researchers.

Moreover, to diminish the malaria prevalence to a low level that does not endanger people’s health and is acceptable to society, the WHO has set targets to manage, eradicate, and control its transmission [13]. The WHO recommends intermittent preventive therapy (IPT) and periodic chemoprophylaxis as the most popular methods to reduce the risk of malaria in high-risk groups such as pregnant women [14]. Long-lived insecticide-treated nets (LLINs), insect larvae control, and indoor insecticide residue spraying (IRS) are techniques to control mosquito vectors [14,15]. Nevertheless, the effectiveness of mosquito repellents may differ depending on the insect vector [15]. To reduce the global disease burden from malaria, an effective malaria vaccine that can be administered in conjunction with existing control initiatives is essential. Now that effective malaria vaccines are being developed, the disease has a stronger likelihood of being eradicated in the 21st century [15]. The most prevalent antigen on the sporozoite surface is the *P. falciparum* circumsporozoite protein (PfCSP), now considered a key target for malaria vaccines [16]. With the upcoming results of additional clinical trials, two primary adjuvanted vaccines against PfCSP are expected to be introduced into immunization programs in malaria epidemic areas [17,18]. Consequently, a number of vaccines related to malaria, including pre-erythrocyte-, erythrocyte-, and transmission-blocking vaccines, are currently being tested in humans [17].

The circum-sporozoite protein (CSP) is the sporozoite’s most abundant surface protein and appears to be a major target of the humoral antibody response. The first licensed malaria vaccine, RTS,S/AS01, which targets pre-erythrocytic malaria, only conferred ~30% protection against clinical malaria in African infants, leaving an ample margin for improvement. Consequently, there is still a need to identify and develop additional malaria vaccines with 75% or greater efficacy against clinical malaria. Several vaccine candidates are currently undergoing testing in human clinical trials, such as those targeting the pre-erythrocytic, erythrocytic, and mosquito stages [17]. The targets of all these vaccines are discussed in this review article.

On the other hand, it is known that schistosomiasis belongs to a group of neglected tropical diseases; it affects 1 billion people and infects approximately 250 million people in mainly 74 different countries worldwide [19]. The trematode that infects the genus *Schistosoma* has a complicated life cycle that may involve the use of aquatic snails as the intermediate host and the use of mammals as the definitive host. Therefore, the most widespread species is *Schistosoma mansoni* (*S. mansoni*) (Patrick Manson, 1902), which is spread by snails, i.e., *Biomphalaria*, and it causes intestinal and liver diseases in many different regions, such as Africa, the Arabian Peninsula, Latin America, and others [20,21]. However, *S. haematobium* (Theodor Bilharz, 1851) is spread by a different snail species, *Bulinus*, and this parasite species causes urogenital schistosomiasis in African countries, including the Arabian Peninsula. Recently, this parasite was also found on the French island of Corsica [22]. The third and last human-infecting *Schistosoma* species, *S. japonicum* (Fujiro Katsurada, 1904), is transmitted by the *Oncomelania* snail, and causes intestinal and hepatosplenic schistosomiasis in various countries, such as the Philippines, China, and Indonesia [23]. It is stated that most of the infections, approximately 90%, occur in African countries, and this schistosomiasis disease causes significant human morbidity and is potentially fatal in several regions [24]. Due to a number of logistical problems, such as poor drug distribution and adherence, sustained reinfection rates, and limited infrastructure, the WHO’s attempts to control schistosomiasis worldwide have yielded less than ideal results [25]. Although these MDA (mass drug administration) programs have been successful in the short term, their long-term viability is questionable. In addition, disruptions to these MDA programs often result in severe rebound disease, particularly in regions of high transmission [26]. PZQ (praziquantel) kills established adult schistosomes, reducing morbidity in treated patients; however, because it is ineffective against juvenile worms and does not prevent reinfection, the overall effect on disease transmission is transient as the prevalence quickly returns to baseline levels after treatment. In the past, vaccine administration was the most economical strategy to prevent long-term multipathogenic human disease. In fact, the introduction of clean water and proper sanitation had an even greater impact on global health than vaccination [25,27]. It is clear that an integrated, comprehensive approach would be needed, with successful vaccination serving as the primary pivot, to achieve sustainable schistosomiasis control goals [25]. We still have a few hurdles to overcome as no vaccine has been approved for human use yet. Nonetheless, some of the positive data on vaccine efficacy from animal and human schistosomiasis challenge models give cause for cautious hope. There are four potential antigens (described below) currently in various stages of clinical development: *Schistosoma mansoni* calpain (Sm-p80/GLA-SE), *Schistosoma mansoni* tetraspanin (Sm-TSP-2), *Schistosoma mansoni* 14-kDa fatty-acid-binding protein (Sm14/GLA-SE), and *Schistosoma haematobium* 28-kD glutathione S-transferase (Sh28GST/Alhydrogel) [28]. Advances in adjuvant technologies also hold promise due to the availability of novel adjuvants capable of selectively activating specific components of the host immune system that are essential for long-term vaccine-mediated immunity. Furthermore, the recently developed controlled human infection model for *Schistosoma mansoni* [29] is likely to accelerate vaccine development and serve as a crucial platform for the identification of new vaccine candidates. The main aspects of schistosomiasis’ biology, epidemiology, management, clinical characteristics, diagnosis, and pathophysiology, and strategies to prevent schistosomiasis disease, have been recently studied [30]. In this comprehensive review article, we consider in detail the vaccine progress regarding schistosomiasis infections and the current state, recent advancements, and main developments in schistosomiasis and malaria vaccines. 

## 2. Survey Methodology

Various scientific search engines such as Science Direct, PubMed, Scopus, etc., were searched and an approximate number of articles published over the last 17 years were retrieved from various scientific literature search engines. Keywords/phrases used to search for relevant data were malaria, schistosomiasis, malaria vaccine, Schistosoma vaccine, and malaria and Schistosoma vaccine status at different clinical stages, reflecting only the relevant literature. Exclusion criteria were also defined. Studies that do not meet the current inclusion criteria, such as thematically irrelevant studies, abstracts, conference proceedings, editorials and comments with insufficient data, were excluded.

### 2.1. Malaria Vaccine

**Figure 1:**
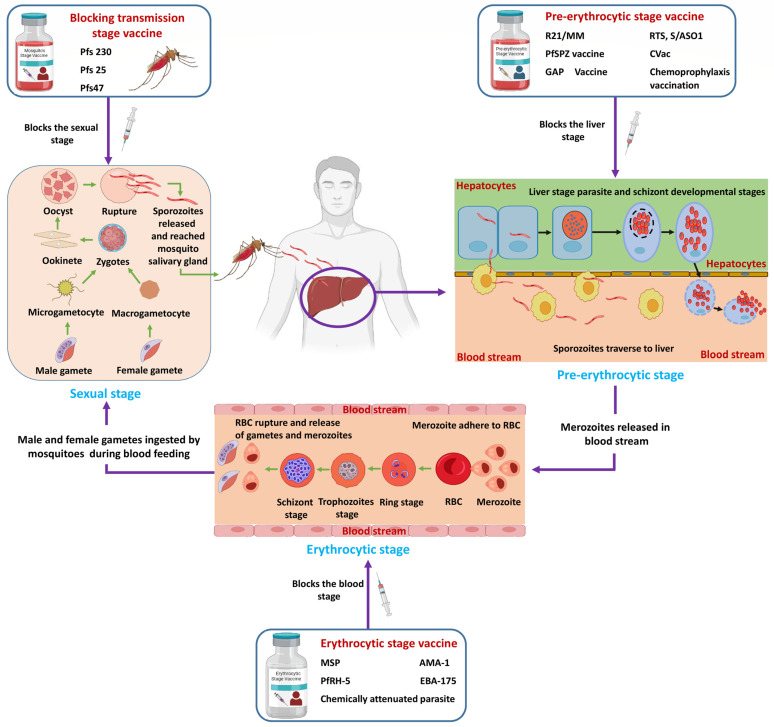
Human malaria vaccines: current state and developments at different stages. Pfs230 (*Plasmodium falciparum* Pfs230 protein); Pfs25 (*Plasmodium falciparum* Pfs25 protein); Pfs47 (*Plasmodium falciparum* Pfs47 protein); R21/MM (R21/Matrix-M™ vaccine); RTS’S/AS01 (repeated T-epitopes derived from PfCSP and S-antigen derived from hepatitis B surface antigen and the AS01 adjuvant); PfSPZ (*Plasmodium falciparum* sporozoite); CVac (chemically modified vaccine); GAP (genetically attenuated parasite); RBC (red blood cell); MSP (merozoite surface protein); AMA-1 (apical membrane antigen 1); PfRH-5 (*Plasmodium falciparum* reticulocyte-binding protein homolog 5); EBA-175 (erythrocyte-binding antigen 175).

A major setback in malaria eradication is the emergence of drug resistance to existing antimalarials and insecticides, along with the frequent presence of asymptomatic and submicroscopic infections in endemic regions, underscoring the need for a highly effective malaria vaccine (Figure 1).

Ongoing efforts have been made to develop an effective vaccine for malaria control. All these efforts have provided a single recombinant vaccine RTS,S/AS01, which also has limitations in its effectiveness [31]. The natural protective immunity against malaria observed in adult individuals repeatedly exposed to the parasite in endemic regions is the basis for the malaria vaccine [32]. The major challenges accompanied by vaccine development are as follows. The extensive genetic variability/polymorphisms in many functional essential genes account for the change in the structure of proteins of a parasite, and this is the first and foremost problem in the identification of a vaccine candidate. Secondly, most of the proteins need to be functionally characterized to be targeted in vaccine studies [33]. In addition, most of the proteins are functionally redundant, so targeting a single protein would not disrupt their processes or the growth of the parasite. During infection, a parasite undergoes various morphological changes, accompanied by modifications in its proteome; therefore, it differentially provokes the effector mechanism, which may be either protective or not [34]. The differential expression of proteins at various stages thus makes it challenging to identify the actual targets that provide protective immunity against malaria [35]. Potential malaria vaccine candidates can target any of the three different stages—the hepatic/pre-erythrocytic stage, the blood stage/erythrocytic stage, or the sexual gametocyte stage (mosquito stage)—of the parasite life cycle (Figure 1 and Table 1) [36].

#### 2.1.1. Pre-Erythrocytic (Liver) Stage

A vaccine targeting the pre-erythrocytic stage is mainly aimed at hampering the invasion process and suppressing the sporozoites’ growth at the hepatic stage, thereby ultimately preventing the disease pathology by limiting the parasites from invading erythrocytes. The initial pre-erythrocyte vaccine trial included immunization with live attenuated sporozoites. In different Phase I trials, vaccination in humans with the irradiated sporozoites affirmed the protection against subsequent infection with *P. falciparum* [36,37]. In contrast, the attenuation of irradiated sporozoites is based on multiple random mutations occurring in sporozoites that ultimately lead to a blockage in liver stage development [38]. The reverse genetics approach has been used to delete the vital pre-erythrocytic stage genes, such as UIS3, UI4, and p36, causing the transgenic parasite’s sporozoites to lose the ability to mature into liver-stage merozoites [39]. The UIS3-deleted sporozoite immunized in three consecutive doses showed protection in the rodent model. Even at a high dosage of sporozoite administration, the protection remained in the immunized mice. Despite successful trials, this approach showed certain limitations, such as the cost of production and feasibility of delivery; moreover, in this case, the requirement of intravenous administration resulted in only 5% efficacy with the intradermal route [40]. In particular, GAPs (genetically attenuated parasites) have been developed, which are more stable and potent. These defined attenuated sporozoites can be produced on a large scale to immunize a large population and prevent malaria [41]. In addition, attenuation at a different pre-erythrocytic stage of development results in innumerable degrees of protection, particularly through the immune response. However, the study demonstrated that the systematic validation of the results of cryopreserved GAPs showed robust viability for more than ten years [41]. In vitro culture systems still need to be developed for the large-scale production of GAPs. The attenuation of GAPs is linked to cell-mediated protection [42]. The concept of GAPs could be translatable to the pathogenic asexual blood stage, as a low dosage also contributes to a higher level of protection. Various genes encoded on the surfaces of sporozoites and merozoites have now been identified, cloned, and expressed in various expression systems, which enable us to progress to the next level of a malaria subunit vaccine. Sporozoites are coated with CSP, which elicits strong antibody- or T-cell-mediated immunity during natural exposure or vaccination, which confers substantial protection in endemic areas. Vaccine studies of the CSP protein show low efficacy and immunogenicity [43,44,45]. Another important vaccine trial targeting the pre-erythrocytic stage, used with different formulations of various adjuvant forms, is EMTRAPE (multiepitope–thrombospondin-related protein), consisting of epitopes of B-cells and CD4+ and CD8+ T-cells from six different pre-erythrocytic proteins fused into TRAP [46]. The most advanced, successful, and only known licensed vaccine, RTS,S/AS01, also targets the pre-erythrocytic stage. RTS,S is a recombinant-protein-based vaccine designed with the NANP repeat region from the central region of the circumsporozoite protein (R) and C terminal with T-cell epitopes (T) fused with the S antigen from the hepatitis B virus (S); this fusion protein is co-expressed with HBsAg (S) in yeast formulated with the adjuvant, AS01 [47]. In Phase III clinical trials at various centers in African countries in which malaria is endemic, it has been reported with ~55% efficacy in treating children at the age group of 5–17 months. Efficacy further decreased to ~31–26% in clinical trials of 6–12-week-old infants [47,48]. Continued efforts are being made further to improve the efficacy and stability of the vaccine. RTS,S has been shown to induce a protective immune response with a high antibody titer and low T-cell response. At the same time, there were also protected volunteers found with low antibodies and higher T-cell responses. Due to the variability in the individual response, it is difficult to link the particular type of response with overall protection [49]. 

##### RTS,S/ASO1 Vaccine

The RTS,S vaccine development approach was first proposed in 1987 by the Walter Reed Army Institute of Research (WRAIR) and the GlaxoSmithKline (GSK) group. The first pilot study was conducted in 2019 in a malaria-endemic region [22]. As far as we know, the RTS,S vaccine is able to target a sequence of four amino acids present on the surface of the *P. falciparum* sporozoite. It is stated that the RTS,S/AS01 vaccine is a combination of repeated T-epitopes (RTS) derived from PfCSP (*Plasmodium falciparum* circum-sporozoite protein) with the S-antigen derived from HBSAg (hepatitis B surface antigen) and the AS01 adjuvant, which is based on liposomes obtained by combining two immune-stimulants, MPL and QS-21, as an adjuvant. RTS,S/AS01 is a recombinant antigen expressed in yeast *Saccharomyces cerevisiae* [22,47]. The CS protein was modified to include the entire C-terminal region of the protein, which contains specific T-cell epitopes required for T-cell response induction. The RTS,S vaccine contains 25% fusion protein RTS and 75% wild-type HBsAg(S) antigen to maximize antigenicity and is only effective against *P. falciparum* disease. Adjuvant systems AS02 and AS01 are used to administer the fusion protein RTSs [50,51]. AS02 is a water-in-squalene emulsion containing monophosphoryl lipid A and saponin from Quillaja Saponaria bark. The AS01 formulation contains monophosphoryl lipid A and Quillaja Saponaria as an immunostimulant component, and the oil-in-water emulsion components are replaced with liposomes. The final vaccine for administration is obtained by reconstituting a lyophilized preparation of RTS,S antigen with AS01 and injecting it intramuscularly [51]. This vaccine is also known as Mosquirix TM. In Ghanaian children, trials were carried out using the RTS,S antigen with the AS01 and AS02 adjuvant systems; the RTS,S antigen with AS01 showed a greater response. Thus, the AS01 adjuvant system was selected for further Phase 3 trials. The Phase 3 randomized controlled trials were conducted involving 15,459 infants (6–12 weeks) and young children (5–17 months) across 11 centers in 7 sub-Saharan African countries: Burkina Faso, Gabon, Ghana, Kenya, Malawi, Mozambique, and Tanzania [22,50]. The study started in March 2009 and continued until January 2011, with 18 months of follow-up study. Vaccine efficacy for all episodes in this population was 55.1%. Vaccine efficacy for severe malaria was found to be 47.3% for children aged 5–17 months old and 34.8% for children aged 6 weeks to 17 months old [52,53]. These initial results confirm the validity of the efficacy rates observed in Phase 2 trials. The vaccine is well tolerated in infants and children. Preliminary results from the Phase 3 study have reported few cases of meningitis and seizures after increasing to seven days of vaccination. Future analyses will determine whether the vaccine response is sustainable over a 32-month follow-up period [22,50,52]. The RTS,S vaccine study was well designed, but it had some limitations: the field study covered only African infants and children, despite the fact that malaria affects a wide range of populations, including those in Asia and South America. Furthermore, the follow-up periods in these studies were short; it is unclear for how long the vaccine retains its efficacy, making it difficult to predict the time intervals required for booster dose administration. In October 2021, the WHO approved the RTS,S/ASO1 vaccine for use in pilot and large-scale studies in children in malaria-endemic areas such as sub-Saharan Africa and other areas with high malaria transmission [52]. One of the main goals of this RTS,S/AS01 vaccine is to eliminate and provide 100% protection from malaria; consequently, the RTS,S/AS01 vaccine is scheduled to enter Phase 4 clinical trials in various malaria-endemic regions to be evaluated. Clinical study results from the RTS,S/AS01E vaccine study were published in 2013, and 447 infants and children aged 5 to 17 months were enrolled in Kilifi, Kenya, for this study. All of these children received three doses of the vaccine (NCT00872963) [22,52,54]. The results showed that the effectiveness of the RTS,S/AS01E vaccine decreased by approximately 44% (95% CI; 16–62) to 0 between the first and fourth subsequent years. However, in 2015, the second clinical trial was conducted and the results of a Phase 3 vaccine trial involving 15,459 children aged 5 to 17 months at 11 sites in 7 sub-Saharan African countries were reported (NCT00866619) [53]. With a 20-month booster dose, the results of the RTS,S/AS01 vaccine showed that targets were only partially met and the vaccine provided partial protection against clinical malaria with a median follow-up of 48 months. However, even after a booster vaccination of this vaccine, there was no change in the children (age group 6–12 weeks) regarding protection against severe malaria [53]. In addition, further studies are ongoing to evaluate the effectiveness of the vaccine in children with four vaccinations. The RTS,S/AS01 vaccine was found to provide better protection against malaria caused by *Plasmodium* species matching the protein used in the vaccine [22,53]. In the following year, 2016, two expert panels, the Strategic Advisory Group of Experts on Immunization (SAGE) and the Malaria Policy Advisory Committee (MPAC), decided not to recommend the RTS,S/AS01 vaccine for general use, pending the results of further clinical studies [22]. However, in July 2015, after the EMA (European Medicines Agency) had received good results and good scientific support for the RTS,S/AS01 malaria vaccine, the WHO recommended several pilot studies on the use of the RTS,S/AS01 vaccine in children aged 5 months in different malaria-endemic locations [49,55]. The fourth vaccine dose was only given between 15 and 18 months after the first three doses, at least 1 month apart [31,49]. The results of clinical trials and mathematical modeling showed that the RTS,S/AS01 vaccine, when used in conjunction with other antimalarial therapies, enhances protection against malaria infection and also showed that the RTS,S/AS01 vaccine has the potential to significantly improve public health [55]. In addition, the study showed that infants from the age of 5 months benefit the most from four doses of the vaccine. Future investigation of these preliminary results in real-world conditions is needed to determine the vaccine’s safety, the mortality implications, and the feasibility of administering four doses in an immunization/vaccination program on a broad basis in different African countries and severely malaria-affected regions [22,55]. In contrast to infection with malaria parasites that have an allelic mismatch, additional research is needed to assess the potential of RTS,S/AS01 vaccines towards different malaria parasites with a PfCSP allele that links with the vaccine target. The only malaria vaccine with ongoing large-scale post-licensing pilot implementation initiatives is currently RTS,S/AS01. Thousands of newborns and children received the RTS,S/AS01 vaccine this year in most malaria transmission countries, such as Ghana, Malawi, and Kenya [22,56].

##### R21/Matrix-M™ Vaccine (R21/MM)

The R21/MM malaria vaccine has elicited researchers’ interest due to the high potency response seen in 2022. It is known that this vaccine is a combination of the R21 virus-like protein based on the PfCSP fused to the N-terminus of HBsAg and combined with the Matrix-M adjuvant [57]. This vaccine is manufactured by Oxford University and the R21/MM vaccine contains Matrix-M, a patented saponin-based adjuvant manufactured by Novavax. The results of an exploratory Phase 2b research study on the human safety and the effectiveness of this malaria vaccine, R21/MM, were published in May 2021 (NCT03896724) [57]. Convincing data from Phase 2b randomized vaccine trials have been obtained in various malaria-endemic areas, such as West Africa, Burkina Faso, and other regions where high seasonal malaria transmission is reported. A study was conducted by two organizations, namely, the Institut de Recherche en Sciences de la Sant (IRSS) and the Nanoro Clinical Research Unit (CRUN). Additionally, this Phase 2b trial included 450 infants and children aged between 5 and 17 months. In this Phase 2b clinical trial, the organization chose to administer three doses of the vaccine at different doses, and children received either a low dose, such as 5μg R21 with 25μg MM, or a high dose of 5μg R21 with 50μg MM, and a Rabies vaccine was used as a control group [57]. Finally, the effects and efficacy of this vaccine were observed after six months, and they found 74% (95% CI; 63–82) efficacy in the low-dose R21/MM adjuvant group and 77% (95% CI; 67–84) efficacy in the high-dose R21/MM adjuvant group [57]. Moreover, no major adverse effects have been reported with R21/MM immunization [22]. However, it was observed that the children who received three doses of the R21/MM vaccine, 28 days apart, had significant antimalarial antibody titers found at the higher adjuvant dose [57]. Moreover, 77% of newborns and children showed good vaccination effectiveness. In addition, a booster dose of the vaccine given one year after the initial three doses of this vaccine as part of an R21/MM immunization regimen can elevate antibody titers to values close to the highest antibody titers [22,57]. Therefore, with regard to the efficacy and safety profile of this human R21/MM vaccine, as well as the ability to maintain immunity in children with annual R21/MM vaccine boosters, optimism about the possibility of eradicating and treating the malaria infection has been renewed [58]. Notably, due to its high R21/MM vaccine efficacy/potency of 77%, this malaria vaccine was the first to reach the 75% efficacy target set by the WHO [54]. The Phase 2b study was extended for a further two years to evaluate the effectiveness of additional booster doses of the malaria vaccine R21/MM by the funding organization [22]. In early 2023, the results of an ongoing clinical study (NCT05252845) to evaluate the immunogenicity and safety of this malaria R21/MM vaccine in adult humans in Thailand will be published [59]. In addition, Oxford University investigators are testing the protection and immunogenicity of this human R21/MM vaccine in adult Thai individuals with the aim of determining whether the concomitant use of different antimalarial drugs has an impact on the immunogenicity of the R21/MM vaccine [59]. Furthermore, in this study, antimalarial drugs such as piperaquine and a single low-dose primaquine treatment will be evaluated for their pharmacokinetics and absorption when co-administered with the R21/MM vaccine [22,59]. It is hoped that adults will experience the high potency and immunogenicity of malaria vaccines seen in infants and children. In addition, in early 2023, we anticipate the completion/receipt of Phase 3 testing results, which are ongoing at scale to verify the safety and efficacy of the vaccine. In addition, in this Phase 3 trial, 4800 infants and children between the ages of 5 and 36 months will be enrolled in four different African countries, namely, Mali, Tanzania, Burkina Faso, and Kenya [60]. Therefore, should the results of these ongoing clinical trials be positive, regulatory approval of this human R21/MM malaria vaccine in 2023 would represent a significant improvement in the safety and health of the world and potentially save the lives of millions of people.

#### 2.1.2. Blood (Erythrocytic) Stage Vaccine

It is the blood stage that is amenable to all disease pathologies during malaria, and natural immunity to malaria is also observed due to many blood-stage antigens. Experiments with the passive transfer of antibodies were able to reduce the disease symptoms and parasitemia in children with malaria infection. The vaccine development strategies propose an approach that can elicit an immune response in children that would also protect adults from disease manifestation [61]. Considering the above, it is speculated that RTS,S might induce immunity to the blood stage by rendering the extended exposure of circulating blood-stage parasites at a lower density [62,63]. Most of the blood-stage vaccine studies focus on the invasion of merozoites, during which the parasites are transiently exposed to the host immune system. The surface proteins of the merozoites are in direct contact with the immune cells, and these proteins are crucial for the parasite’s growth as well; therefore, these proteins are considered candidates in developing a blood-stage vaccine [64,65]. SPf66 was the first blood-stage asexual malaria vaccine to be developed. It contains a multi-stage peptide vaccine mixed with alum as an adjuvant, but further development of this vaccine has been halted because numerous Phase III studies have shown that the efficacy of this vaccine is very low [66]. Various vaccine candidates are merozoite surface proteins found to produce an effective immune response in preclinical trials [67]. Some of the well-studied vaccine candidates under clinical trials are apical membrane antigen 1 (AMA1), serine repeat antigen 5 (SERA5), ring-infected erythrocyte surface antigen (RESA), erythrocyte-binding antigen 175 (EBA175), merozoite surface protein (MSP)-1, MSP-2, MSP-3, erythrocyte-binding antigen 175, and glutamate-rich protein (GLURP), which are all exposed to the surfaces of the blood-stage antigens [67,68,69]. The technological challenges of generating *Plasmodium* proteins in a recombinant form that is biochemically active is one of the main problems arising from the use of recombinant proteins for the production of blood-stage vaccines. The functional redundancy between the merozoite ligands involved in erythrocyte invasion is a significant obstacle to the creation of a highly effective blood-stage malaria vaccine. Merozoites are thought to have an assemblage of overlapping ligands expressed on their surfaces, giving the parasite access to multiple invasion pathways. In order to effectively prevent a wide range of invasion routes, it is believed that effective blood-stage vaccination should target different merozoite antigens simultaneously [69]. The large allelic variation observed in many *Plasmodium* strains is a significant obstacle to the creation of an effective blood-stage malaria vaccine, in addition to the parasite’s ability to gain access to multiple invasion routes. The two foremost blood-stage vaccine candidates, AMA1 and MSP142, were not protective in African adolescents, partly because of antigenic differences. Despite sufficient antibody titers, cross-protection against different malaria strains was insufficient [70,71]. However, it was recently discovered that RH5 and basigin interact in a way that is generally required for erythrocyte invasion, raising new expectations for the development of a very effective blood-stage vaccination [72]. Low levels of anti-basigin monoclonal antibodies can completely prevent erythrocyte invasion; this effect has been observed in numerous laboratory-adapted *P. falciparum* lines and field isolates [72,73]. Conversely, in nine separate *P. falciparum* strains harboring all five of the most common RH5 polymorphisms, polyclonal antibodies produced against the 3D7 variant of RH5 were able to prevent erythrocyte invasion [74]. In addition, RH5 delivered via a virus can elicit antibodies that prevent a variety of parasite genetic variations from entering erythrocytes. Therefore, it is expected that the discovery of the interaction between RH5 and Basigin will lead to the creation of effective therapeutic approaches targeting the blood stages of the parasite. The first Phase Ib results of the VV-RH5 immunization and the RH5.1 protein vaccine in African infants are therefore eagerly awaited by researchers (ClinicalTrials.gov: NCT03435874 and NCT04318002) [75]. These results will be crucial in determining whether the CHMI overestimated the effectiveness of RH5-based vaccines in adults in the UK due to the significantly weaker antibody response. To further increase the effectiveness of RH5-based vaccines, the other recently identified antigens, CyRPA and RIPR, which combine with RH5 to form a heterotrimeric protein complex, could be included. Similar to RH5, these antigens are extremely conserved, necessary, and capable of inducing significant levels of anti-growth antibodies following animal vaccination [72,76,77]. Finally, it is crucial to consider logical adaptations to the structure and mode of delivery of the RH5 immunogen. Two methods to achieve large increases in the quantity and/or quality of vaccine-induced polyclonal anti-RH5 IgG include the increased delivery of innovative RH5 immunogen arrays on virus-like particles and the analysis of vaccine-induced human anti-RH5-mAbs to inform structure-based vaccine design [75,76,77,78]. To guide these forthcoming methods of rationally improving vaccination, we require a thorough study of the RH5 antibody epitope specificities evoked by the RH5.1/AS01B vaccine, coupled with structural and functional outcomes [79].

#### 2.1.3. Transmission-Blocking Vaccine

Vaccines that block transmission are not intended to provide direct protection; rather, the aim is to inhibit the formation of gametocytes, which would ultimately lower the transmission rate and thereby reduce the rate of infection [80]. The transmission-blocking vaccines interfere with the sexual stage of the parasite. Earlier experiments on malaria models have reported malaria transmission-blocking immunity. When turkeys or chickens are vaccinated with extracellular male or female gametes or gametocytes of the malaria parasite, the animals develop immunity that may eventually protect them from further infection with the same parasite by infected mosquitoes. Based on this concept, mosquitoes are allowed to feed on the blood of rodents and humans, containing antibodies against the gametocytes, ookinete, or zygote [80]. These antibodies will attack the respective stage of antigens and block the development of the parasite to further stages. Many vaccine trials for antigens have been initiated. However, the most promising antigens are Pfs-48/45 and Pfs-230 of *P. falciparum*’s and their orthologs in *P. vivax*, expressed on both macrogametes and microgametes [81]. Pfs-48/45 and Pfs-230 antigens have a 6-cysteine structure, represented in the human host [81]. These proteins contain polymorphic sequences and have heavily conserved domains. Antigens Pfs-25 and Pfs-28 are expressed in zygotes and on the matured ookinetes of the malaria parasite [82]. These proteins are abundant in cysteines such as the Pfs-12 family of proteins, but the arrangement of cysteines is different, having four complete 6-cysteines or four partial cysteine EGF-like domains [83].

Therefore, the results from human clinical trials of two transmission-blocking antigen (TBV) vaccine candidates, Pfs230 (pre-fertilization antigen) and Pfs25 (post-fertilization antigen), were obtainable [84]. According to related studies, it was found that human immunity against sex-stage malaria parasites can act in the mosquito. On the other hand, NIAID researchers coupled both Pfs25 and Pfs230 to the immunogenic carrier protein exoprotein (EPA) and administered adjuvants to overcome the antigens’ inherently low immunogenicity [84]. Furthermore, human clinical studies of Pfs25-EPA performed with Alhydrogel demonstrated the safety and immunogenicity of the antigen in humans. In addition, functional antibodies can be induced by the antigen in laboratory tests to prevent parasite transmission to mosquitoes. Serum activity also showed a correlation with antibody titers [85]. However, the majority of vaccine recipients required four doses to produce functional serum activity, and this activity was transient as the anti-Pfs25 antibody levels fell rapidly after each dose [86,87]. Remarkably, the titers against the carrier protein EPA decreased more slowly than the titers against Pfs25 IgG. Ongoing studies are associating and merging the vaccine antigens Pfs25 and Pfs230 [84]. Standard membrane feeding assays (SMFAs) and the direct feeding of mosquitoes to vaccine recipients are both used to measure vaccine efficacy. Since Pfs230 vaccination is well recognized for the complement to be reliant on, the SMFA requires a new complement. GSK’s AS01 adjuvant is being used in an ongoing experiment with both the Pfs230 and Pfs25 conjugate human vaccines, as pre-clinical studies indicate that this formulation could dramatically increase antibody titers and consequently lead to efficient serum activity following immunization [87,88,89,90]. Overall, the research group has completed the first clinical trial and demonstrated its safety and immunogenicity in an alum-adjuvanted formulation of the Pfs25 vaccine. Thus, the serum from these subjects had a macerating but suboptimal transmission-blocking effect, on the order of a 50% reduction in the infectivity of *P. falciparum* to mosquitoes compared to controls [88,89,90]. 

### 2.2. Schistosoma Vaccine

Efforts to combat schistosomiasis have been significant, largely due to population-based blocking chemotherapy provided by MDA and regular oral PZQ administration, targeting primarily at-risk school-age children with no history of diagnosis [91]. Indeed, the ability of sustained PZQ treatment to reduce schistosomiasis morbidity worldwide has led to a significant (and perhaps excessive) focus on chemotherapy as the only way to completely eradicate the disease [92]. Despite this development, the prevalence of schistosomiasis remains largely unaffected since rapid reinfection after each regular MDA treatment quickly brings infection rates back to pre-existing levels [92]. Of further note is the fact that PZQ is not 100% successful, and, in particular, each course of treatment involves only a single dose, resulting in the untreated migrating *schistosoma* growing into the next cohort of adult worms (1–2 months), according to the MDA guidelines [93,94]. Although there is currently no direct, conclusive evidence that clinically relevant drug-resistant schistosomes have evolved here, the possibility of PZQ drug resistance developing as a result of the widespread use of the treatment is an ongoing concern [94,95].

A significant reduction in prevalence has only been achieved in a few schistosomiasis-endemic regions, particularly in China, where elimination is now expected. However, this benefit can be attributed to other factors besides the use of PZQ, such as good snail management, environmental changes, and health education [94]. In this context, it must be noted that species of snails outside of Southeast Asia that serve as intermediate hosts are not amphibious and are therefore much more difficult to manage. As a result, it is now almost universally recognized that the effective prevention and control of schistosomiasis requires a multi-pronged, integrated strategy. This situation underscores the need to develop and deploy a vaccine as a key component of an offensive strategy to offset the very short-lived effects of the drug/chemotherapy [96]. In fact, based on feasibility and need, a schistosomiasis vaccine is one of the 10 most important vaccines that need immediate and successful development [25]. Consequently, an alternative that would offer a crucial long-term feature for use in conjunction with MDA is the creation and placement of an efficient vaccine within a disease control spectrum. It is stated that this vaccine (schistosomiasis) may well not even be 100% effective according to advanced mathematical modeling, supporting the idea that even partially protective vaccination could help to reduce/block schistosomiasis transmission/infection [97,98].

It is regrettable that no licensed/commercial schistosomiasis vaccine is presently available in the market against any strain of the *schistosoma* parasite, underscoring the scientist/researcher’s requirement for constant determination to achieve this elusive target. However, due to funding issues and the complex immunology pathway involvement as the human host vacillates between fighting off an impending infection and escaping significant pathology when responding to trapped tissue eggs from previous infections, researchers in this field have struggled for many years [98]. Schistosomes have also developed sophisticated survival tactics over the course of their evolution, allowing them to successfully move between multiple, wildly different conditions to achievement entry into the human host. Once researchers ceased participating, it became increasingly difficult to create a good product, which limited the possibility of rapidly reaching the goal. The fact that schistosomes do not replicate in their intended hosts offers some hope as opposed to viral and bacterial infections, so there is some potential for successful treatment in this respect. Therefore, even minimally effective vaccination could help to reduce infection rates and stop the spread of the disease, supporting a previous hypothesis presented by different models, such as mathematical ones [99]. It has been suggested that the main mechanism being sought may not be an immune pathway or immune system lead in the case of the mature schistosome worm [100]. If this assumption is correct, the parasite could be most susceptible to an immune response during the short window of time between the invasion of the cercarial skin and the appearance of schistosomula in the lungs, which occurs around 72 h later [100]. Since the juvenile schistosome in the hepatic sinusoids is already immune to complement- and antibody-dependent cellular cytotoxicity (ADCC), this susceptibility is likely to be at least partially transferred there [101].

#### 2.2.1. Current Human Clinical Vaccine

There are four major lead human-based schistosomiasis vaccines (Table 2) that are undergoing clinical trials at various development phases, and they are Sm-p80, Sm-TSP-2, Sh28GST, and Sm-14.

##### Sh28GST Vaccine

The Sh28GST (recombinant *S. haematobium* 28 kDa glutathione S-transferase) vaccine with Alhydrogel (aluminum hydroxide), called Bilhvax, was developed in collaboration with researchers from Eurogentec, Lige, Belgium and the team of Professor Andre Capron from the Inserm University of Lille, developed with the Institute Pasteur de Lille in France [102]. In a Phase 1 human clinical trial, the Sh28GST vaccine was shown to be immunogenic, safe, and able to produce a Th2-type immune response in children and male adults. However, although the rSh28GST+Alhydrogel vaccine demonstrated robust, safe, and long-lasting immunogenicity and anti-worm fecundity with reduced egg viability, the main objective of the clinical trial study, a significant delay in schistosomiasis recurrence, was not met [103]. Importantly, the selection factors/criteria promoting the conservation or modification of functionally compelling amino acid residues of schistosomal GST may reflect a modification in how the host’s immune system identifies the parasite. According to clinical studies that showed an unusually high level of development of its conserved N-terminal domain, it is believed that schistosomal 28 kDa GST no longer functions as an enzyme but rather as a target of the host’s immune system, specifically to induce immunity reactions, affecting parasite fitness [103]. It is claimed that there are multiple amino acid repeats of surface proteins that result in highly polymorphic molecules known as smoke screens, mimicking a parasitic approach to ineffectively awaken the host’s immune system [91]. Species with a shortened phylogenetic gap, such as Sb (*Schistosoma bovis*) and Sh (*Schistosoma haematobium*), and a large phylogenetic gap, such as Sm (*Schistosoma mansoni*) and Sj (*Schistosoma japonicum*), were found to have found altered evolutionary arrangements in the N- terminal domain of the molecule. Furthermore, all these *Schistosoma* species show approximately 2-fold variance in the number of non-synonymous nucleotide substitutions, indicating low immunogenicity [28]. However, researchers found that within natural schistosomal parasite populations, polymorphisms in immunogenic regions of GST can impair its suitability as a vaccine candidate [104]. Natural history research, therefore, is particularly important in regions where hybrid schistosomes, which are the subject of therapeutic studies, are known to be endemic. Assessing the basic immunological and genetic population profiles and the above-mentioned exposure to parasites such as *Schistosoma* species and other co-infectious agents is mandatory as they can influence the vaccine response and efficacy against *Schistosoma* parasites [28]. Furthermore, it is well understood that these infections and co-infections can alter immune pathways and regulation, and the present study suggests that individual infection affects susceptibility, severity, and immunological responses to subsequent infections. Overall, this encouraging result has motivated investigators/scientists to further test the rSh28GST vaccine as a potential vaccine candidate against urogenital schistosomiasis [28]. The effectiveness of the rSh28GST vaccine could be increased, which could motivate further clinical tests. This could be achieved by changing the experimental design and/or using a diverse adjuvant to achieve a more stable isotypic response while reducing the number of vaccine administrations.

##### Sm-14

It is known that Dr. Miriam Tendler and her research partner have long worked to develop a recombinant 14-kDa fatty-acid-binding protein (FABP) from *S. mansoni* (rSm-14), which is important for *Schistosoma* uptake, transport, and the compartmentalization of host-derived protein sterols [104]. To facilitate the uptake of lipids from the host, Sm-14 protein enzymes are located in the cytosol and in tissues adjacent to the interfaces of parasite/host contact, e.g., the basal lamina of the worm tegument and muscle layers [105,106]. They are also present in the intestinal epithelium, where they are used by the parasite for the transfer and utilization of lipids. In addition to mediating ADCC, Sm-14-specific antibodies bind to the dorsal surface tubercles of the parasite and prevent the uptake of lipids necessary for the parasite’s survival. It was claimed that Sm-14, along with the *S. mansoni* homologue of Sh28GST, was one of the original panels of putative *S. mansoni* vaccine antigens proposed by WHO/TDR in the late 1980s [106]. This Phase 1 study of the rSm-14 vaccine was completed in 2011, and this phase evaluated the safety and immunogenicity of the rSm-14 vaccine. The researcher used a GLA adjuvant, which was used in the formulation of the rSm-14 vaccine [107,108]. In addition, the pilot study was conducted in the Brazilian state of Rio de Janeiro and the results showed that no major side reactions were detected during testing on 20 male volunteers from a non-endemic schistosomiasis region. However, the most interesting observation of this study is that the vaccine is immunogenic and does not have a high/elicited specific IgE response, which is an optimistic result for this Sm-14 vaccine [108]. In addition, to verify the immunogenicity and safety of the rSm-14 vaccine in humans, a study was conducted on 10 healthy female volunteers, and this study was successfully completed in 2012 [109,110]. Afterwards, the success of the rSm-14 vaccine paved the way for the clinical Phase 2a trial to be carried out in humans in 2015–2017. During this clinical study, the researchers recruited 30 adult males who all belonged to a highly endemic area for *S. mansoni* and *S. haematobium* and received the rSm-14 vaccine [109,111]. Based on the interesting findings in adult males, a Phase 2b study design and scientifically ethical procedure was developed in 2018 that was employed in 95 Senegalese school children between the ages of 7 and 11 in the same region, where both *S. mansoni* and *S. haematobium* occur predominantly [100,112]. Therefore, these groups plan to conduct Phase 2c, 2d, and 3 clinical trials in Senegal and Brazil, respectively.

##### Sm-TSP-2

The third vaccine based on *S. mansoni* tetraspanin (TSP) is currently in Phase 1 clinical testing. It has been found that TSP proteins are most abundant in the outermost tegumental membrane of *Schistosoma mansoni* at different stages in mammals and are persistently exposed to the human immune system [113,114]. Furthermore, researchers have explained in detail that Sm-TSP-1 and Sm-TSP-2 are required for tegumental biogenesis and integrity in the parasite. An interesting result of this vaccine in the mouse model was the high immunity correlated with IgG antibodies with protective immunity in individuals who are inherently resistant. In addition, this vaccine was selected for clinical testing as a lead schistosomiasis vaccine candidate and is currently being manufactured as a 9-kDa Sm-TSP-2/Alhydrogel recombinant vaccine adjuvanted with GLA-SE, via a product development partnership with the Sabin Vaccine Institute [114,115]. The vaccine candidate has completed the required toxicity studies and has shown good pre-clinical results. In addition, many human clinical trials have been conducted or are ongoing with the Sm-TSP-2 vaccine in various areas, such as non-endemic and endemic communities [95]. Specifically, a Phase 1b human clinical trial of this vaccine is ongoing in a region of Brazil where *Schistosoma* species have high transmission availability to evaluate the immunogenicity and safety of the vaccine in a group of healthy volunteers who may have previously been infected with or exposed to the schistosomiasis disease. Further field trials in Uganda are planned [95,115,116].

##### Sm-p80

The tegument of mature schistosomes, as well as at various stages of the parasite life cycle, expresses abundant calpain, a calcium-activated neutral protease [117,118]. Bloodborne helminths utilize tegument biogenesis and/or transformation as a means to evade and/or modulate their host immune responses. Sm-p80 is the large subunit of *S. mansoni* calpain [119]. Sm-p80 has been identified as a viable candidate for schistosomiasis vaccination because of its critical role in schistosomiasis survival in its hostile host environment. A number of efficacy studies have been conducted with Sm-p80-based vaccines over the last 20 years [106]. Therefore, the Sm-p80 vaccine was tested in different animal models, such as mouse, hamster, and baboon models, to show the efficacy and potency of the vaccine using different Sm-p80 vaccine formulations, including naked DNA, recombinant protein, and prime/boost [120]. In addition, the Sm-p80 vaccine has been shown to provide remarkable protection against *S. mansoni* challenge. Furthermore, infected individuals from Africa and South America did not display Sm p80-specific IgE, reducing the possibility of a post-vaccination hypersensitivity reaction [106]. Moreover, Sm-p80 has been shown to have a therapeutic effect in vaccinated baboons by reducing the number of adult worms, decreasing the retention of eggs in tissues, and decreasing the number of eggs that the host sheds in feces [120,121]. Schistosomiasis is significantly killed by a complement after receiving the Sm-p80 vaccine. The administration of PZQ prior to receiving the Sm-p80 vaccine has been shown to significantly reduce tissue retention and hatchability in non-human primates [25,121]. Sm-p80/GLA-SE has now received approval for a Phase I clinical trial as a result of positive responses and results from studies in rodents and non-human primates. Moreover, Phase 1 human clinical trials for the recombinant Sm-p80/GLA-SE vaccine SchistoShield have been initiated [25].

## 3. Challenges for Malaria and Schistosomiasis Vaccines

The CDC has emphasized that numerous obstacles, such as the lack of a traditional market, a lack of developers, and the technical difficulties involved in developing a vaccine against a parasite, needed to be overcome to reach this point. The complex immunological response to malarial infection is poorly understood, and malarial parasites have a complicated life cycle [122,123]. The genetic complexity of malaria parasites produces thousands of possible antigens [124]. Current solutions have only a 65% success rate against severe malaria, work poorly in older children or adults, require a unique adjuvant that reduces supply, and lose most of their protective effects in young children over the first 18 months. The RTS,S exclusively affects the *Plasmodium* parasite species known as *P. falciparum* [124,125]. Protective immunity also progressively deteriorates over time. This indicates that repeated booster doses are required. Alternative vaccination schedules are also being considered. On the other hand, heterogeneity in the vaccination response can occur when schistosomiasis vaccine candidates progress from Phase I, II, and III trials to testing in target populations in different endemic settings because of the differences in disease exposure, co-infections, and transmission conditions that specifically shape the immunological profiles [126]. Therefore, immunological complexity poses unique hurdles for vaccine development and testing in countries where schistosomiasis is endemic, in addition to existing challenges related to logistics, accessibility, and limited resources. While many vaccine candidates have been identified, few have progressed into clinical trials, and they may not provide the level of protection needed. To develop and test novel vaccine antigens with high throughput, an innovative and successful anti-schistosome vaccine pipeline is urgently needed. This pipeline should leverage cutting-edge technologies to identify and target the most suitable vaccine candidates, leverage the mRNA vaccine platform of the highly successful COVID-19 mRNA vaccines, and use the appropriate animal models for immunological analysis and vaccine efficacy testing.

## 4. Conclusions

The development of malaria and schistosomiasis vaccines is a complex and challenging process, and there are several factors that need to be considered in order to ensure that the vaccines are both safe and effective. For example, the *Plasmodium* and *Schistosoma* parasites have a highly complex life cycle, and it is essential to target multiple stages of the parasite in order to induce a strong and lasting immune response. Additionally, the parasite is able to evade the host immune response through the expression of variant antigens, and this must be taken into account when developing vaccines. Another important factor to consider is the age-dependent efficacy of malaria and schistosomiasis vaccines. Young children and infants are particularly vulnerable to severe malaria and chronic schistosomiasis, and it is essential that vaccines that are both safe and effective in these age groups are developed. In addition, the presence of pre-existing immunity to malaria and schistosomiasis in many populations must also be taken into account when developing vaccines, as this can impact the efficacy of the vaccine. The development of a malaria and schistosomiasis vaccine is also subject to regulatory requirements, and the vaccine must undergo rigorous testing and clinical trials to demonstrate its safety and efficacy before it can be licensed for widespread use. This process can be time-consuming and costly. Despite the progress being made in malaria and schistosomiasis vaccine development, there are still significant challenges that need to be addressed. One of the main challenges is ensuring that the vaccine provides long-term protection against the disease. Another challenge is ensuring that the vaccine is affordable and accessible to those who need it most, particularly in resource-limited settings where malaria and schistosomiasis are endemic. In conclusion, the development of effective and accessible malaria and schistosomiasis vaccines is leading to the achievement of global malaria and schistosomiasis elimination worldwide. While there have been some promising results (RTS,S and R21/MM for malaria and Sm-14, Sm-TSP-2, and Sm-p80 for schistosomiasis) in clinical trials, much work remains to be carried out to ensure that effective malaria and schistosomiasis vaccines become available for those who need them. This will require sustained commitment from the scientific and public health communities, as well as increased investment in research and development.

## Figures and Tables

**Table 1:** Malaria vaccine development status regarding pre-erythrocytic, erythrocytic, and sexual stages.

Malaria Parasite Vaccine	Vaccine Categorization	Current Vaccine Status
**Liver stage (pre-erythrocytic)**		
R21/MM	Adjuvanted protein vaccine (virus-like particles based on the PfCSP strain NF54 fused to the N-terminus of HBsAg)	Phase III
RTS, S/ASO1	Subunit	Licensed
PfSPZ vaccine	Whole organism (radiation attenuated)	Phase II
CVac	Whole organism (chemically attenuated)	Phase I
GAP vaccine	Whole organism (genetically attenuated)	Phase I
**Blood stage (erythrocytic)**		
MSP	Subunit	Preclinical
AMA-1	Subunit	Preclinical
PfRH-5	Subunit	Phase Ib
Chemically attenuated parasite	Whole organism	Preclinical
**Transmission blocking antigen (mosquito stage)**		
Pfs230	Subunit	Phase I
Pfs25	Subunit	Phase I
Pfs47	Subunit	Preclinical

**Table 2:** Schistosomiasis vaccine development and current status.

*Schistosoma* Parasite Vaccine	Vaccine Categorization	Current Vaccine Status
Sh28GST	Recombinant 28-kDa glutathione S-transferase from *S. haematobium*	Phase III
Sm-14	Recombinant 14-kDa (rSm-14) *S. mansoni* fatty-acid-binding protein	Phase III
Sm-TSP-2	Recombinant Sm-TSP-2/Alhydrogel vaccine	Phase Ib
Sm-p80	Recombinant	Phase I

## Data Availability

Not applicable.
